# The National Institute of Mental Health: Research Agenda and Priorities in Geriatrics and Aging

**DOI:** 10.1093/geroni/igaa057.2115

**Published:** 2020-12-16

**Authors:** Elizabeth Necka

**Affiliations:** National Institute of Mental Health, Rockville, Maryland, United States

## Abstract

The Geriatrics and Aging Processes Research Branch of the National Institute of Mental Health (NIMH) supports research on the etiology, pathophysiology, and trajectory of late life mental disorders. The branch encourages research using neuroscience, cognitive and affective science, and social and behavioral science to translate basic and preclinical research to clinical research. The branch prioritizes research that investigates neuropsychiatric disorders of aging, how they interact with neurodevelopment/neurodegeneration, and how to assess, treat, and prevent them. Of particular interest is research on social isolation and suicide. Suicide prevention research is an urgent priority: NIMH’s portfolio includes projects aimed at identifying those at risk for suicide, understanding causes of suicide risk, developing suicide prevention interventions, and testing the effectiveness of these interventions and services in real-world settings. In this talk, a NIMH program official will discuss the NIMH research agenda in the domain of late-life mental illness, social isolation, and suicide.

